# Rp58 and p27^kip1^ coordinate cell cycle exit and neuronal migration within the embryonic mouse cerebral cortex

**DOI:** 10.1186/s13064-017-0084-3

**Published:** 2017-05-15

**Authors:** Olivier Clément, Isabel Anne Hemming, Ivan Enghian Gladwyn-Ng, Zhengdong Qu, Shan Shan Li, Michael Piper, Julian Ik-Tsen Heng

**Affiliations:** 1 The Harry Perkins Institute of Medical Research, Perth, WA 6009 Australia; 2 The Centre for Medical Research, University of Western Australia, Perth, WA 6009 Australia; 3 EMBL Australia, The Australian Regenerative Medicine Institute, Monash University, Clayton, VIC 3800 Australia; 4 The School of Biomedical Sciences, University of Queensland, Brisbane, 4072 Australia; 5 Queensland Brain Institute, University of Queensland, Brisbane, 4072 Australia; 6 Curtin Health Innovation Research Institute, Curtin University, Bentley, 6845 Australia

**Keywords:** Cerebral Cortex, Transcription Factor, Neurodevelopment, Neurogenesis, Radial migration, Neuronal morphology

## Abstract

**Background:**

During the development of the mammalian cerebral cortex, newborn postmitotic projection neurons are born from local neural stem cells and must undergo radial migration so as to position themselves appropriately to form functional neural circuits. The zinc finger transcriptional repressor Rp58 (also known as Znf238 or Zbtb18) is critical for coordinating corticogenesis, but its underlying molecular mechanism remains to be better characterised.

**Findings:**

Here, we demonstrate that the co-expression of Rp58 and the cyclin dependent kinase inhibitor (CDKI) p27^kip1^ is important for E14.5-born cortical neurons to coordinate cell cycle exit and initiate their radial migration. Notably, we find that the impaired radial positioning of *Rp58*-deficient cortical neurons within the embryonic (E17.5) mouse cortex, as well as their multipolar to bipolar transition from the intermediate zone to the cortical plate can be restored by forced expression of p27^kip1^ in concert with suppression of *Rnd2*, a downstream target gene of Rp58. Furthermore, the restorative effects of p27^kip1^ and *Rnd2* abrogation are reminiscent of suppressing RhoA signalling in *Rp58*-deficient cells.

**Conclusions:**

Our findings demonstrate functional interplay between a transcriptional regulator and a CDKI to mediate neuroprogenitor cell cycle exit, as well as to promote radial migration through a molecular mechanism consistent with suppression of RhoA signalling.

**Electronic supplementary material:**

The online version of this article (doi:10.1186/s13064-017-0084-3) contains supplementary material, which is available to authorized users.

## Introduction

The development of the cerebral cortex involves a precise integration of multiple molecular cues within embryonic cortical cells to coordinate the production of appropriate numbers of progenitor cells, followed by the elaboration of distinct neural cell types [1–4]. Within the developing embryonic cerebral cortex, local progenitor cells undergo multiple rounds of cell proliferation to generate neurons, astrocytes and oligodendrocytes which systematically integrate within the tissue so as to eventually form functional neural circuitry. The activities of DNA-binding transcription factors have been recognised to be crucial for guiding the developmental trajectory of embryonic cortical neurons through the initiation of cell-intrinsic programmes which specify their subtype as excitatory, glutamatergic neurons or inhibitory, GABAergic neurons [3, 5–7]. For example, the proneural bHLH transcriptional activator Neurogenin2 (Neurog2) drives a cell intrinsic programme to specify an excitatory glutamatergic neuron fate. Furthermore, Neurog2 initiates the transcription of downstream target genes, including the Rho GTPase related factor *Rnd2*, which specifies radial positioning of newborn neurons within the growing cortical anlagen [8, 9]. Equally, the activities of transcriptional repressors, such as the zinc finger DNA binding transcriptional repressor Rp58 (also known as Znf238 or Zbtb18), are also critical to the development of the embryonic cerebral cortex. Notably, the gene regulatory activities of Rp58 influence the timing of Neurog2 expression within the embryonic cortex to promote neurogenesis [10], and also directly regulates *Rnd2* expression for the efficient radial migration of newborn cortical neurons [11]. As a corollary, loss of Rp58 expression during embryogenesis leads to neurodevelopmental defects such as premature depletion of cortical progenitors, precocious neurogenesis and gliogenesis, as well as programmed cell death [10, 12–14].

In addition to transcription factors, members of the Cip/Kip family of cyclin dependent kinase inhibitor (CDKI) proteins are also critical for coordinating neuroprogenitor cell cycle exit and differentiation within the developing cortex [12, 15–17]. Notably, the CDKI p27^kip1^ drives neuroprogenitor cell cycle exit and cortical neuron differentiation through its cyclin kinase inhibitor functions [15–17], while it also mediates neurite outgrowth through its capacity to suppress RhoA signalling so as to coordinate the neuronal cytoskeleton [16]. More recently, p27^kip1^ has also been identified to promote microtubule polymerisation to facilitate the migration of cortical cells [18]. While such findings identify critical roles for transcription factor signalling and CDKI activity during cortical neurogenesis, their cooperative functions remain less well characterised, particularly given recent evidence linking Rp58 expression and CDKI activities in the development of astrocytes [12]. Here, we report a functional relationship between Rp58 and p27^kip1^ to drive cell cycle exit and promote distinct phases of radial migration during cerebral cortex development.

## Methods

### Animals

Mice (C57BL/6 J) were housed, bred and treated within the animal facilities at Monash University. Female mice of at least 6 weeks of age were utilised for timed-matings. *Rp58*-knockout mice were genotyped by polymerase chain reaction. All animal procedures are approved by the Animal Ethics Committee within Monash University (Licenses MARP/08–104 and MARP/2012/068), and are compliant with guidelines provided by the National Health and Medical Research Council of Australia.

### DNA plasmids and Antibodies

Mammalian (pCaggs) expression vectors encoding p27^kip1^, p27^kip1^(ck-) and RhoA(N19) have been described previously [16], while an Rp58 expression construct with a pCIG vector which lacks a GFP cassette was used [11]. RNAi for *Rp58* was achieved using a pool of targeting siRNAs (Dharmacon GE Life Sciences) which was previously verified for specificity of knockdown as well as a pSilCaggs-*Rnd2*shRNA1 vector to induce *Rnd2* RNAi [11]. Primary antibodies used for immunostaining analysis include chicken antibody to GFP (Abcam, ab13970, 1:700), mouse anti-p27^kip1^ (BD Biosciences, 1:400), rabbit anti-Rp58 (Proteintech Group, 1:250), rabbit anti-Ki67 (NCL-Ki67p, Leica, 1:1000), pHH3(ser10) (06–570, Merck Millipore, 1:1000), mouse anti βIII-tubulin (Covance, MMS-435P, 1:1000), mouse anti-Nestin (Millipore, MAB353, 1:300), rabbit anti-Pax6 (Covance, PRB-2788, 1:500), rabbit anti-Tbr2 antibody (Abcam, ab233345, 1:500), rabbit polyclonal antibody to GFP (Invitrogen, A6455, 1:1000). Alexa fluor secondary antibodies include goat anti- chicken IgG (Invitrogen, A11039, 1:700), goat anti-mouse (Invitrogen, A11031, 1:800), and goat anti-rabbit IgG (Invitrogen, A6455, 1:1000). The nuclei of cells were visualised with DAPI.

### *In Utero* Electroporation

*In utero* electroporation experiments are performed as described [19, 20]. High quality, low endotoxin plasmid preparations (Qiagen) of DNA vectors were injected at 1 μg/μl for each plasmid, together with Fast Green (0.05%, Sigma). For RNAi experiments, Dharmacon siRNA targeting pools were injected at 10 μM concentration together with GFP expression plasmid at 1 μg/μl concentration. Following recovery from *in utero* electroporation, the mice were sacrificed by cervical dislocation, and the embryonic brains were harvested by dissection in cold PBS. For studies of dissociated cortical cells, dissected embryonic cortical tissue was digested to obtain a single-cell suspension and plated as per previously described [9]. For histological analysis, electroporated brains were subject to fixation in 4% paraformaldehyde solution in PBS overnight followed by three washes in PBS and permeation in 20% sucrose/PBS solution. Following tissue embedding in OCT, cryosectioning along the coronal plane (16 μm thickness) was performed followed by fluorescence immunostaining for antigens of interest. Images of brain sections were captured on an epifluorescence microscope (Olympus) equipped with a CCD camera (SPOT). Subdivisions of the embryonic cortex (VZ/SVZ, IZ and CP) were identified based on cell density as visualised with DAPI (4′6-Diamidino-2-Phenylindole) staining, as described previously [21]. Images from embryonic E17.5 cortices for cell shape analyses were acquired at ×20 magnification, as per described previously [22]. Cell counting was performed blind to the condition on representative fields of sections of electroporated brains using ImageJ software.

### Cell Culture, Western blotting and immunoprecipitation

Mouse embryonic carcinoma (P19) cells were cultured in Dulbecco’s modified Eagle’s medium (Gibco 10,313) supplemented with 10% heat-inactivated fetal bovine serum (Thermo Fisher HYC15–010.02), 2 mM L-glutamine (Gibco 25,030), 20 units/ml of penicillin and streptomycin (Gibco 15,140) under humidified air containing 5% CO2 at 37 °C. Transfections were performed using equal quantities of expression plasmids (pcDNA empty vector, pcDNA-Neurog2 or pcDNA-Rp58 vector) for each condition. Western blotting analysis was performed with antibodies to Rp58, actin (A5441, Sigma Aldrich) and p27^kip1^ together with appropriate fluorescent secondary antibodies, as described [22]. Immunoblot signals were resolved detected with an Odyssey® infrared imaging system (Li-Cor 9201–02) for analysis.

## Results

**Fig. 1:**
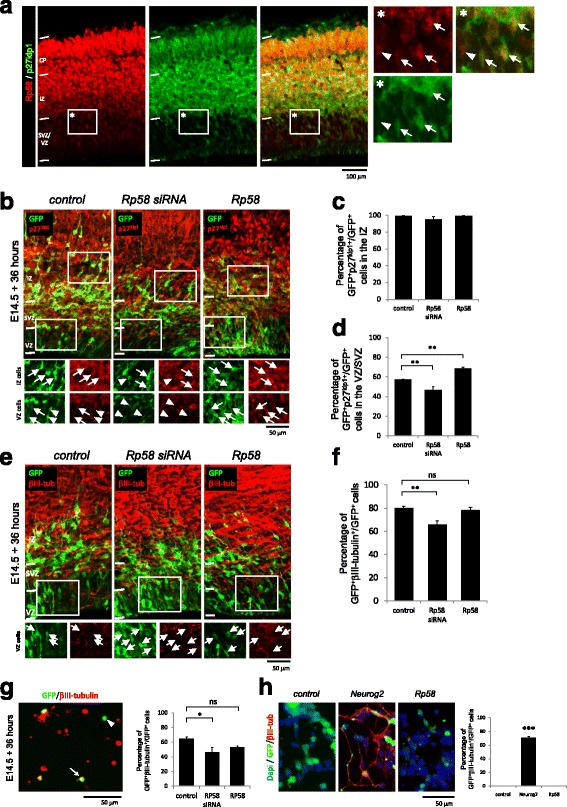
The effects of *Rp58* disruption on p27^kip1^ within the embryonic cortex. **a** Immunostaining of Rp58 and p27^kip1^ to reveal their co-presence in cells of the VZ/SVZ, IZ and CP. Arrows point to double-positive cells, while arrowheads point to Rp58^+^ cells. **b**–**d** Knockdown or overexpression of Rp58 did not significantly disrupt p27^kip1^ co-immunostaining of GFP+ cells in the IZ (F_2,6_ = 3.1 *p* = 0.11, One-way ANOVA, >300 cells counted from 3 independent brains per condition) (**c**), while there was a significant effect in the VZ/SVZ (F_2,6_ = 45, *p* < 0.0005, One-way ANOVA, >400 cells counted from 3 independent brains per condition). Arrows point to double-positive cells, while arrow heads point to GFP^+^ cells. **e**, **f** Knockdown of *Rp58* leads to a decrease in the proportion of GFP electroporated cells which co-label with βIII-tubulin, indicative of impaired neurogenesis (F_2,6_ = 18 *p* = 0.003, One-way ANOVA, >600 cells counted from 3 independent brains per condition). Arrows point to GFP+ cells which do not co-label with βIII-tubulin. **g** GFP labelled cells were dissociated from successfully electroporated brain tissue and plated for 2 h before fixation, immunostaining and data collection. Knockdown of Rp58 leads to a reduction in neurogenesis, while forced expression does not significantly disrupt p27^kip1^ expression (F_2,6_ = 9 *p* = 0.011, One-way ANOVA, >450 cells counted from 3 independent experiments per condition). Arrow points to double-positive cells, while arrow head points to GFP^+^ cells.. (H) Forced expression of Neurog2 but not Rp58 in P19 embyrocarcinoma cells induces neurogenesis, as evaluated by immunostaining for βIII-tubulin. *p < 0.05, **p < 0.01, ***p < 0.001; “ns” denotes not significant. Scale bar represents 50 μm

We focussed our attention on early-mid gestation mouse embryos at embryonic day 14.5 (E14.5), a stage of neurodevelopment defined by a peak period of neurogenesis when maximal numbers of cortical neurons are born from local ventricular zone (VZ) and subventricular zone (SVZ) progenitor cells [23]. We performed immunostaining and detected Rp58 in neuroprogenitor cells of the ventricular zone (VZ) and subventricular zone (SVZ), as well as in postmitotic neurons of the intermediate zone (IZ) and cortical plate (CP) (Fig. 1a). We also characterised the immunostaining pattern for Rp58 together with p27^kip1^, the predominant cyclin dependent kinase inhibitor in the E14.5 embryonic cortex [16] and detected their co-expression in scattered cells within the germinal VZ/SVZ, and in virtually all cells of the IZ and CP (Fig. 1a). Since it is known that disruptions to Rp58 or p27^kip1^ lead to defective neuronal differentiation and radial migration [10, 11, 13, 16], we reasoned that the combined activities of both Rp58 and p27^kip1^ might also be relevant for coordinating the cellular activities of cortical progenitors, such as their cell cycle exit and radial migration.

We performed Western blotting with lysates collected from whole E14.5 embryonic cortices of *Rp58* wildtype, heterozygote and nullizygous embryos to find that steady-state levels of p27^kip1^ immunoblotted signal were not significantly different between genotypes (Additional file 1: Figure S1A-B; F_2,7_ = 0.26, *p* = 0.77 One-way ANOVA). While this result is consistent with the findings of Hirai and colleagues [12], we were intrigued by the possibility that cell-type specific fluctuations in p27^kip1^ within the cortical tissue might underlie *Rp58* deficiency. Thus, we performed a series of experiments using E14.5 mouse embryos in which we introduced small interfering RNAs (siRNAs) by *in utero* electroporation to efficiently suppress *Rp58* in a cell autonomous manner within the embryonic cortex, as previously reported [11]. We also performed control experiments in which cortices were electroporated with a non-targeting siRNA pool. In these experiments, a Green Fluorescent Protein (GFP) expression vector was co-electroporated to identify siRNA-treated cells. In parallel, we also electroporated brains with a bicistronic GFP expression construct encoding RP58 (together with non-targeting siRNAs) to investigate the consequence of its forced expression within the E14.5 cortex. Thirty-six hours after electroporation, successfully electroporated brains were analysed for potential effects on p27^kip1^ expression. As shown in Fig. 1b, we found that neither knockdown of *Rp58* nor its forced expression led to a significant effect in the proportion of GFP-labelled cells which co-stain with p27^kip1^ in the IZ (Fig. 1c; F_2,6_ = 3.1, *p* = 0.11, One-way ANOVA, >300 cells counted from 3 independent brains per condition). We also analysed the intensity of p27^kip1^-immunofluorescence signal within IZ cells and found no significant difference between treatment groups (Additional file 1: Figure S1C-D; F_2,222_ = 2.9, *p* = 0.0557, One-way ANOVA, >75 cells counted from 3 independent brains per condition). However, we observed a significant reduction in the proportion of p27^kip1^-expressing cells within the VZ and SVZ following *Rp58* knockdown, while overexpression led to a significant elevation (Fig. 1b, d; F_2,6_ = 45, *p* < 0.0005, One-way ANOVA, >400 cells counted from 3 brains per condition). Furthermore, the intensity of p27^kip1^-immunofluorescence signal within VZ/SVZ cells was significantly reduced upon knockdown, while overexpression did not have a significant effect (Additional file 1: Figure S1E; F_2,222_ = 95, *p* < 0.0001, One-way ANOVA, >75 cells counted from 3 independent brains per condition). These observations suggest that acute suppression of *Rp58* results in a transient reduction in p27^kip1^ levels in VZ/SVZ cells, with potential effects on neurodifferentiation. Consistent with this finding, *Rp58* knockdown led to a significant reduction in neuronal differentiation as marked by immunostaining for βIII-tubulin, but forced expression of Rp58 did not have such an effect (Fig. 1e, f, F_2,6_ = 18, *p* = 0.003, One-way ANOVA, >600 cells counted from 3 brains per condition). Parallel experiments in which electroporated tissue was dissociated and cells plated before immunostaining for βIII-tubulin further confirmed this finding (Fig. 1g, F_2,6_ = 9, *p* = 0.011, One-way ANOVA, >450 cells counted from at least 3 brains per condition). While our results could also be interpreted to indicate that Rp58 drives neurodifferentiation, we addressed this directly in an experiment using mouse embryocarcinoma P19 cells to find that forced expression of Rp58 does not promote the production of βIII-tubulin expressing neurons *in vitro* (Fig. 1h, F_2,6_ = 1212, *p* < 0.001, One-way ANOVA, >1000 cells counted from 3 independent experiments per condition). As a control, we transfected cells with the neurogenic transcription factor Neurog2 which robustly induced βIII-tubulin expression. Altogether, these results show that disruptions to *Rp58* affect p27^kip1^ expression in embryonic cortical cells of the germinal VZ/SVZ, but Rp58 lacks the capacity to promote neurodifferentiation.

**Fig. 2:**
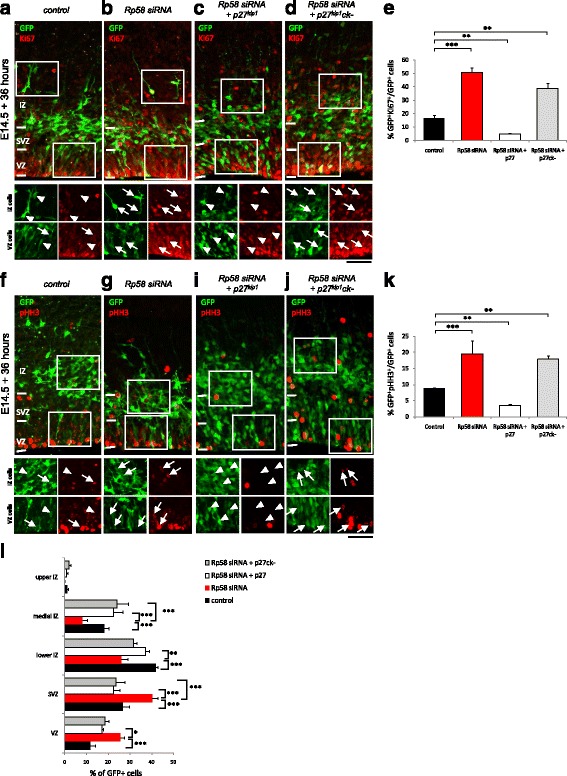
p27^kip1^ restores the defective cell proliferation and radial migration of *Rp58* siRNA-treated cortical progenitors. Knockdown of *Rp58* leads to significant reductions in the expression of the cell proliferation marker Ki67. **a**–**d** The defective expression of Ki67 in *Rp58* siRNA-treated cells could be restored with p27^kip1^, but not p27^kip1^(ck-) which is incapable of signalling cell cycle exit owing to a mutation which impairs its cyclin kinase function (**e**) (F_3,8_ = 73, *p* < 0.001, One-way ANOVA, >700 cells counted from 3 independent brains per condition). Similar effects on the co-detection of pHH3, a marker of cell mitosis, were observed (**f**–**k**, F_2,8_ = 20, *p* = 0.004, One-way ANOVA, >700 cells counted from 3 independent brains per condition). **l** In addition, suppression of *Rp58* by siRNA treatment impaired the migration of GFP-labelled cells, while treatment with either p27^kip1^ or p27^kip1^(ck-) promoted the radial migration of *Rp58*-siRNA treated cells from the VZ/SVZ to the IZ (F_2,8_ = 12, *p* < 0.0001, One-way ANOVA, >550 cells counted from 3 independent brains per condition). Scale bar represents 50 μm

We previously reported that Rp58 cell autonomously regulates progenitor cell cycle exit and initiation of radial migration within the E14.5 embryonic cortex [10, 11]. Given our findings that Rp58 is co-labelled with p27^kip1^ within the embryonic E14.5 cortex (Fig. 1a), and that disruptions to Rp58 lead to changes in p27^kip1^ expression within the VZ/SVZ (Fig. 1b–d; Additional file 1: Figure S1C-E), we hypothesised that Rp58 mediated neuroprogenitor proliferation via a p27^kip1^-mediated process. Should this be the case, we would thus predict that forced expression of p27^kip1^ might abrogate Rp58 deficiency in neural progenitor cells. To test this, we co-delivered expression constructs encoding p27^kip1^ or a variant which lacks its cyclin kinase function but retains its capacity for signalling cell migration (termed p27^kip1^ck-) in *Rp58*-deficient cortical cells of the E14.5 cortex and examined their potential restorative effects on cell proliferation and radial migration. It is known that p27^kip1^ promotes cell cycle exit through its activities involving cyclin dependent kinases as well as cell migration through a mechanism independent of its cyclin kinase activity [16, 24], and we confirmed this effect in the E14.5 cortex (Additional file 2: Figure S2). As shown in Fig. 2, while *Rp58* siRNA treatment leads to a significant increase in the proportion of GFP-labelled cells which co-label with the proliferation markers Ki67 (Fig. 2a–d, and Fig. 2e, f_3,8_ = 73, *p* < 0.001, One-way ANOVA, >700 cells counted from 3 independent brains per condition) or pHH3 (Fig. 2k; F_2,8_ = 20, *p* = 0.004, One-way ANOVA, >700 cells counted from 3 independent brains per condition), co-delivery of p27^kip1^ led to a significant restoration of these markers, while co-delivery of a p27^kip1^ck- expression construct did not have a restorative effect. Notably, p27^kip1^ suppressed cell proliferation to an extent beyond control levels (Fig. 2e, Ki67 16.6% ± 2.1% in control vs 4.9% ± 0.6% in *Rp58* siRNA restored with p27^kip1^ expression; *p* < 0.01), suggesting that p27^kip1^ and Rp58 have different potencies for mediating Ki67 and pHH3 expression in cortical cells. We also found a significant interaction between non-surface (SVZ) divisions in treated cells identified as mitoses marked by pHH3 expression away from the ventricular surface (Additional file 3: Figure S3A; F_3,9_ = 7, *p* < 0.0102, One-way ANOVA, 3 independent brains per condition), which could suggest an effect of Rp58 disruption on neuroprogenitor cells. To investigate this further, we analysed the proportion of Pax6-expressing radial glial progenitors to find that this was not significantly affected between treatment groups (Additional file 3: Figure S3B-F; F_3,9_ = 0.89, *p* = 0.4818, One-way ANOVA, >600 cells counted from 3 to 4 independent brains per condition). We also did not find a significant difference in surface versus non-surface positioning of GFP + Pax6+ cells (data not shown). In contrast, treatment with *Rp58* siRNAs led to a significant reduction in the proportion of Tbr2-expressing intermediate progenitors which could not be augmented by co-delivery of p27^kip1^ or p27^kip1^ck- expression constructs (Additional file 3: Figure S3G-K; F_3,11_ = 6.5, *p* = 0.0085, One-way ANOVA, >600 cells counted from 3 to 4 independent brains per condition). Overexpression of p27^kip1^ or p27^kip1^ck- alone did not influence Pax6-immunoreactive radial glial progenitors, while only p27^kip1^ overexpression resulted in a reduction in Tbr2-immunopositive cells (Additional file 2: Figure S2E-F). Thus, the effect of *Rp58* knockdown on intermediate progenitors cannot be restored by forced expression of p27^kip1^ or p27^kip1^ck-.

**Fig. 3:**
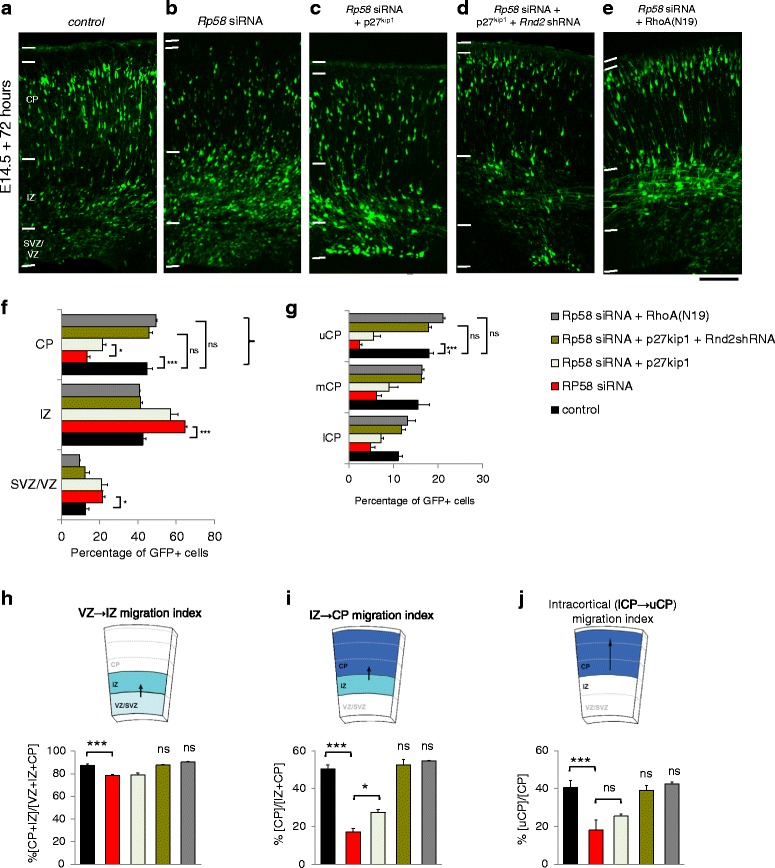
The defective migration of *Rp58*-deficient cells can be restored with forced expression of p27^kip1^ and concurrent suppression of *Rnd2* by RNAi, and this phenotype is reminiscent of *Rp58*-deficient cells co-treated with a dominant-negative RhoA(N19) expression construct**. a–e** Representative images of GFP-labelled cells within the embryonic cortex following treatment with the conditions indicated. **f** There was a significant interaction between treatment condition and distribution of GFP-labelled cells (F_8,36_ = 106, *p* < 0.0001, Two-way ANOVA, >700 cells counted from 3 to 4 independent brains per condition). Knockdown of *Rp58* with siRNAs leads to significant decrease in migration into the CP (**a**, **b**, **f**; *p* < 0.0001 Bonferroni posthoc *t*-test), but CP entry is only modestly restored by co-delivery with p27^kip1^ (**b**, **c**; **f**; *p* < 0.01 Bonferroni posthoc *t*-test). Forced expression of p27^kip1^ alone promotes radial migration into the CP (Additional file 6: Figure S5A–D). However, only co-delivery of p27^kip1^ and *Rnd2* shRNA leads to a restoration of the migration of *Rp58* siRNA-treated cells (**b**, **d**; **b**; *p* < 0.0001 Bonferroni posthoc *t*-test) to levels not significantly different to control CP profile. The radial migration of *Rp58*-deficient cells can also be restored by co-delivery of RhoA(N19) (**b**, **e**; **f**; *p* < 0.0001 Bonferroni posthoc *t*-test), and the CP profile is not significantly different to control. **l** Quantification of the proportion of GFP-expressing cells within each subcompartment of the embryonic (E17.5) cortex. **g** There was a significant interaction between treatment condition and distribution of GFP-expressing cells within the lower, medial and upper cortical plate (lCP, mCP and uCP, respectively) (F_8,33_ = 9.0, *p* < 0.0001, Two-way ANOVA, >700 cells counted from 3 to 4 independent brains per condition). **h** The VZ to IZ phase of migration calculated across conditions. **i** The IZ to CP phase of migration calculated across conditions. **j** The lower CP to upper CP phase of migration calculated across conditions. One-way ANOVA followed by Bonferroni’s posthoc *t*-test; *p < 0.05, **p < 0.01, ***p < 0.001 for (**h**) to (**j**). Scale bar represents 100 μm

During cortical development, immature neurons undertake distinct migratory behaviours as they leave the germinal VZ to migrate through the IZ so as to position themselves appropriately within the CP. We previously reported that *Rp58*-deficient cells are impaired in their capacity for radial migration within the embryonic cortex [10, 11]. However, p27^kip1^ is known to promote the migration of neurons into the embryonic cortical plate ([16] and Additional file 2: Figure S2G), thus we hypothesised that both factors could signal cooperatively to promote radial migration. Curiously, in addition to the restorative effects of p27^kip1^ over-expression on cell cycle exit in *Rp58*-deficient cortical cells (Fig. 2a–k), we also observed that co-delivery of either p27^kip1^ or p27^kip1^ck- augmented their migration from the VZ/SVZ to the IZ (Fig. 2l; F_2,8_ = 12, *p* < 0.0001, One-way ANOVA, >550 cells counted from 3 independent brains per condition). Guided by this finding, we explored whether forced expression of p27^kip1^ could restore the defective migration of *Rp58*-deficient neurons into the CP of the embryonic mouse cortex. We thus performed *in utero* electroporation experiments with E14.5 embryos in which we delivered *Rp58* siRNAs together with p27^kip1^ and examined treated brains 72 h later. Previously, we reported that suppression of *Rp58* expression using siRNAs in E14.5-born cortical cells impaired their capacity to migrate into the CP of the embryonic E17.5 cortex, and that this defect was restored by co-delivery of an Rp58 expression construct which was refractory to silencing [11]. As shown in Fig. 3, while *Rp58*-siRNA treated cells were defective in their migration into the CP, co-delivery of p27^kip1^ led only to a modest increase in the proportion of cells in the CP (Fig. 3a–c, f). Furthermore, we examined the distribution of GFP-labelled cells within the CP to find that co-delivery of p27^kip1^ did not correct the intracortical positioning of *Rp58*-deficient cells (Fig. 3a–g). However, we have also previously reported that Rp58 regulates radial migration through transcriptional regulation of downstream target genes such as *Rnd2*, and that the defective migration of *Rp58*-deficient cells could be partially corrected by abrogating *Rnd2* expression [10, 11]. Therefore, we reasoned that the mechanistic actions for Rp58 to signal radial migration within the cortex could involve both p27^kip1^, as well as transcriptional regulation of *Rnd2*. When we directly tested this hypothesis by co-delivering *Rp58* siRNAs with p27^kip1^ as well as a short hairpin RNA expression vector with moderate silencing activity for *Rnd2* (known as *Rnd2*shRNA1, see [11]), we found that the radial migration of these cells was significantly augmented, such that their migration into the CP (Fig. 3f, 44.66% ± 2.79% in the CP for control treatment versus 46.01% ± 2.59% in the CP for *Rp58* siRNA + p27^kip1^ + *Rnd2*shRNA1 treatment, *p* > 0.05 Bonferroni posthoc *t*-test) as well as their intracortical positioning (Fig. 3g, 17.99% ± 1.01% in the upper CP for control treatment versus 16.09% ± 0.81% in the upper CP for *Rp58* siRNA + p27^kip1^ + *Rnd2*shRNA1 treatment, *p* > 0.05 Bonferroni posthoc *t*-test) was restored to levels not significantly different to control profile. This was further confirmed when we analysed the phases of cell migration within each embryonic cortical subcompartment, namely the VZ to IZ migration (Fig. 3h, F_4,12_ = 39, *p* < 0.001 One-WAY ANOVA), IZ to CP migration (Fig. 3i, F_4,12_ = 99, *p* < 0.001 One-WAY ANOVA) and lower CP to upper CP migration (Fig. 3j, F_4,12_ = 19, *p* < 0.001 One-WAY ANOVA). As shown, while *Rp58* siRNA-treatment impaired migration across all compartments, co-delivery of both p27^kip1^ and *Rnd2* shRNA led to a significant improvement in all phases of migration by *Rp58*-deficient cells to levels not significantly different to control profile (Fig. 3h–j).

It has been reported that both p27^kip1^ and Rnd2 mediate cell migration within the embryonic cortex by suppressing RhoA signalling [16, 25]. Thus, we extended our investigation to determine if the defective migration of *Rp58*-deficient cells could be restored by co-delivery of an expression construct encoding a dominant-negative form of RhoA, (denoted RhoA(N19) [26]). As shown, forced expression of RhoA(N19) significantly augmented the migration of *Rp58* siRNA-treated cells to a profile which was not significantly different to control treatment in the CP (Fig. 3a–f; 44.66% ± 2.79% in the CP for control treatment versus 49.53% ± 0.35% in the CP for *Rp58* siRNA + RhoA(N19) treatment, *p* > 0.05), as well as within the upper CP (Fig. 3g; 17.99% ± 1.01% in the upper CP for control treatment versus 21.15% ± 0.44% in the upper CP for *Rp58* siRNA + RhoA(N19) treatment, *p* < 0.05 Bonferroni posthoc *t*-test). Furthermore, treatment with RhoA(N19) corrected all phases of migration (Fig. 3h–j). We performed immunostaining with Nestin antibody to confirm that the radial glial scaffold of the embryonic cortices between treatment groups was not significantly perturbed (Additional file 4: Figure S4), and that our tissue specimens were prepared in a consistent manner. Therefore, these studies indicate that Rp58 mediates cell migration through p27^kip1^ and Rnd2 via a cell autonomous mechanism which phenocopies the suppression of RhoA signalling.

**Fig. 4:**
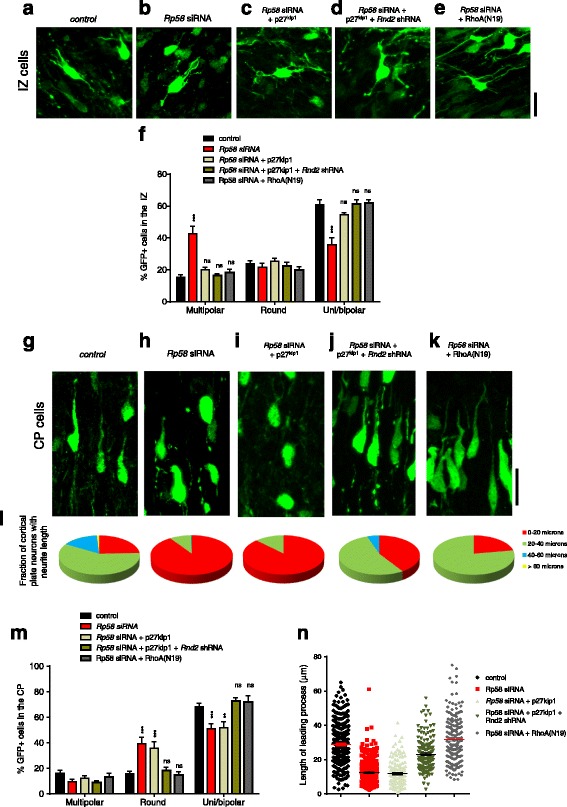
The effects of p27^kip1^ and *Rnd2* abrogation on the morphology of *Rp58* siRNA-treated embryonic cortical neurons. **a**–**e** Representative images of IZ neurons from each treatment group. **f** There is a significant interaction between the distribution of round, uni/bipolar and multipolar cell shapes and treatment condition (F_8,60_ = 20, *p* < 0.0001, Two-way ANOVA followed by Bonferroni’s posthoc *t*-test, >300 cells counted from 3 to 4 independent brains per condition), with a significant increase in the proportion of multipolar cells and a concomitant decrease in uni/bipolar shaped cells upon *Rp58* siRNA treatment (*p* < 0.001 Bonferroni posthoc *t*-test). **g**–**k** Representative images of CP neurons from each treatment group. **l** Pie charts plotting distribution of the lengths of leading processes of uni/bipolar shaped neurons within the CP for each treatment condition. **m** There is a significant interaction between the distribution of round, uni/bipolar and multipolar cell shapes and treatment condition (F_8,60_ = 12, *p* < 0.0001, Two-way ANOVA followed by Bonferroni’s posthoc *t*-test, >300 cells counted from 3 to 4 independent brains per condition), with a significant increase in the proportion of round cells and a concomitant decrease in multipolar shaped cells upon *Rp58* siRNA treatment (*p* < 0.001 Bonferroni posthoc *t*-test). **n** The average lengths of leading process of uni/bipolar shaped neurons is decreased upon *Rp58* siRNA treatment, and this is restored by co-treatment with p27^kip1^ and *Rnd2*shRNA1 or RhoA(N19) (F_4,1187_ = 211, *p* < 0.0001, One-way ANOVA, >160 neurons analysed per condition). Scale bars represent 20 μm

Radial migration by cortical neurons involves a series of dynamic changes to their morphologies which, in turn, culminate in directional movement as multipolar-shaped neurons or as bipolar-shaped cells [2, 27]. As newborn neurons leave the germinal VZ/SVZ, they migrate as bipolar shaped neurons then transit as multipolar-shaped neurons within the IZ. Finally, these neurons undergo multipolar-to-bipolar transition, engage a radialglial fibre and then migrate to their appropriate position within the cortical plate by locomotion. We performed high power confocal microscopy on GFP-labelled cortical cells within the IZ and CP in each of our treatment conditions in order to understand how modulation of Rp58, p27^kip1^ and Rnd2 expression influenced their morphology. We found that knockdown of *Rp58* resulted in an accumulation of multipolar-shaped cells with very short processes interspersed amongst round-shaped cells within the intermediate zone (Fig. 4a–f), consistent with previous reports [10, 11, 25]. Forced expression of p27^kip1^ significantly restored the morphological profiles of *Rp58*-deficient cells to control levels within the IZ, suggesting that p27^kip1^ and Rp58 may share common functions for mediating the shapes of IZ cells (Fig. 4a–f). However, within the CP compartment, forced expression of p27^kip1^ could not restore the elevated proportion of round cells and concomitant reduction in uni/bipolar shaped neurons as a consequence of *Rp58* siRNA treatment (Fig. 4g–i, m). Furthermore, we found that while knockdown of *Rp58* results in a significant reduction in the length of the leading processes of CP neurons, forced expression of p27^kip1^ also could not restore this feature (Fig. 4 g–i, l, n). In contrast, however, treatment with both p27^kip1^ and *Rnd2* shRNA restored the morphological profiles of *Rp58*-deficient neurons to levels not significantly different to control treatment (Fig. 4f and m for IZ and CP cell shape distributions, respectively), suggesting that p27^kip1^ and Rp58 (through suppression of *Rnd2*) are both required to regulate distinct morphological characteristics of embryonic cortical neurons over the course of their radial migration. Furthermore, we observed that the morphological profiles for *Rp58*-deficient cells co-treated with RhoA(N19) were also restored to levels not significantly different to control treatment (Fig. 4 f–n). Thus, our findings suggest that the defective morphological properties of *Rp58*-deficient cells can be corrected by augmenting p27^kip1^ levels in combination with suppression of *Rnd2*, and that these restorative effects on cell shape are reminiscent of a molecular mechanism involving suppression of RhoA signalling.

## Discussion

In this study, we have revealed cooperative functions for Rp58 and the cyclin dependent kinase inhibitor p27^kip1^ during the development of the embryonic mouse cerebral cortex. Our results indicate that suppression of Rp58 leads to a transient loss of p27^kip1^, and that forced expression of p27^kip1^ restores the defective cell cycle exit and radial migration of *Rp58*-deficient cortical cells from the germinal VZ/SVZ to the IZ. Furthermore, we find that the defective CP migration of *Rp58*-deficient cells could only be restored by p27^kip1^ in combination with down-regulation of *Rnd2* by RNAi, and that the cellular features of these neurons are consistent with a mechanism involving down regulation of RhoA signalling. Thus, our findings support a role for Rp58 and p27^kip1^ to jointly coordinate cell cycle exit and the early (VZ/SVZ-to-IZ) phase of cell migration within the developing cerebral cortex, while the capacity for cortical cells to undergo the late (IZ-to-CP) phase of migration involves distinct functions mediated by p27^kip1^ and Rp58-Rnd2 signalling pathways (see Additional file 5: Figure S6).

Rp58 deficiency during embryogenesis leads to profound defects in cortical development, including cell proliferation, differentiation and tissue morphogenesis [10, 12–14, 28]. However, a precise understanding of its mechanistic actions on the development of distinct neural cell types is only beginning to emerge. A recent study found that loss of Rp58 led to aberrant neuroprogenitor proliferation and precocious gliogenesis through a molecular mechanism involving de-repression of *Id* genes which, in turn, resulted in the elevation of steady state levels of the CDKI p57^kip2^ in the embryonic cortex [12]. However, while Ids are known to influence the expression of multiple CDKIs [29–31], levels of p27^kip1^ were not significantly disrupted in Rp58 heterozygous and homozygous cortices [12]. Crucially, our findings provide evidence that *Rp58* deficiency leads to a transient loss in p27^kip1^ expression, with consequences to cell cycle exit, neurogenesis and radial migration likely through multiple signals (see summary diagram in Additional file 5: Figure S6). In neuroprogenitors, Rp58 directly represses the cell cycle regulatory genes *Id1–4* [12], and Id3 is a repressor of *p27* ^*kip1*^ [29]. Based on our findings, we entertain the hypothesis that *Rp58* deficiency results in defective cell cycle progression attributable to de-repression of Id3 in VZ/SVZ cells (see Fig. 2 of [12]) which, in turn, leads to a transient loss of *p27* ^*kip1*^ within this sub-compartment of the embryonic cortex. However, the subsequent restoration of p27^kip1^ in IZ cells is more difficult to explain, and remains to be clarified in future investigations. Regardless, our data is consistent with the notion that Rp58 and p27^kip1^ signal cell cycle exit through distinct pathways, since we observed that forced expression of p27^kip1^ in *Rp58*-deficient cells resulted in the suppression of proliferation markers Ki67 and pHH3 to levels beyond control treatment.

As cortical neuroprogenitors undergo cell division, p27^kip1^ drives cell cycle exit and these cells subsequently express the proneural transcription factor Neurog2 so as to specify their identity as excitatory, glutamatergic neurons [4, 32]. Also, Neurog2 initiates a gene regulatory programme for cell migration which involves the transcriptional activation of downstream target genes including *Rnd2* [5, 33, 34]. In parallel, the transcriptional repressor activity of Rp58 is critical for negatively regulating *Neurog2* expression in cortical cells to coordinate timing of neurogenesis [10]. Crucially, p27^kip1^ stabilises Neurog2 protein so as to promote neurodifferentiation [16], and so our current findings suggest that the defects in cortical neurogenesis as a consequence of *Rp58*-deficiency is also attributable to a transient fluctuation in p27^kip1^ levels. This, in turn, would negatively influence Neurog2 protein levels and ultimately disrupt proneural transcriptional regulation in embryonic cortical cells. Our findings are therefore consistent with a model which reconciles the transcriptional regulatory functions for Rp58 with an epistatic mechanism for cell cycle exit involving signalling cooperativity with p27^kip1^ (Additional file 5: Figure S6).

In contrast to the epistatic effects of Rp58 and p27^kip1^ on cell proliferation, we find that treatment with *Rp58* siRNAs led to a reduction in expression of the intermediate progenitor marker Tbr2, and that forced expression of p27^kip1^ or p27^kip1^ck- was not restorative for this defect. While an investigation to establish the underlying molecular mechanisms for these effects is beyond the scope of our present study, our result is in contrast to a previous finding that Rp58 nullizygous mouse cortices display elevated numbers of Tbr2-immunopositive cells [10, 13]. We believe that these observations point to distinct effects on intermediate progenitors which differ between acute loss of *Rp58* expression following siRNA treatment (reported in our study and in [11]) versus chronic loss of Rp58 in homozygous mutant (Rp58^−/−^) mice [10, 13]. A recent study in which the authors performed *in utero* electroporation of E14.5 cortices to introduce cre recombinase reported no significant effect on Tbr2 immunostaining in labelled cells 24 h after treatment [10]. However, it was reported that cre electroporation did not lead to complete loss of Rp58 protein expression after 24 h, which is likely to explain the lack of effect on Tbr2 expression. Collectively, these data altogether demonstrate a temporal effect of Rp58 disruption on the development of cortical neuroprogenitors during embryonic development.

In newborn postmitotic neurons, Rp58 negatively regulates appropriate levels of *Rnd2* within migrating cortical neurons so as to guide cells to their appropriate positions within the cortex [10, 11]. We previously reported that Rp58 regulates radial migration through *Rnd2*, but we also recognised that additional signals were at play, since abrogation of *Rnd2* expression was not sufficient to fully restore the defective migration of *Rp58*-deficient cells [11]. Based on the results from our current study, we find that the defective, early (VZ/SVZ to IZ) phase of migration of *Rp58*-deficient cells can be restored by co-delivery of p27^kip1^, while the late (IZ to CP) phase of migration can only be restored by manipulation of both p27^kip1^ and Rp58-Rnd2 signalling. While we acknowledge that our results are based on gain-of-function approaches to augment p27^kip1^ in cells, we believe they are sufficient to draw the conclusion that Rp58 influences CDKI activity in VZ/SVZ cells to drive neuroprogenitor cell cycle exit and migration into the IZ, while parallel signals from a CDKI such as p27^kip1^ combine with Rp58-Rnd2 signalling for the appropriate positioning of neurons within the CP. Presently, it is recognised that forced expression of p57^kip2^ restores the defective proliferation of E14.5-treated cells of *Rp58*-nullizygous mouse cortices [12]. However, knockdown of p57^kip2^ impairs the migration of E14.5-born mouse cortical neurons [35], yet Nguyen and colleagues reported that forced expression of p57^kip2^ did not significantly enhance migration [16]. Taken together, given the potencies of both CDKIs p27^kip1^ and p57^kip2^, our study is consistent with the notion that p27^kip1^ is a crucial, physiologically-relevant effector for coordinating neurodifferentiation and cell migration in concert with Rp58 within the E14.5 embryonic cortex.

Cell motility is orchestrated by the activities of small GTPases, including RhoA and Rac1, in order to form a leading process and to retract the trailing process, respectively, for directional movement [36]. In fibroblasts, RhoA signalling is down-regulated by p27^kip1^ so as to enable cells to limit the formation of stress fibres and focal adhesions which can impair their motility [24]. Similarly, p27^kip1^ is found to downregulate RhoA signalling in cortical neurons to facilitate their radial migration [16]. While these lines of evidence indicate that RhoA signalling is relevant to migration, appropriate levels of Rnd2 are also crucial to down regulate RhoA signalling during this process [9, 25]. In our study, we find that the defective migration of *Rp58*-deficient neurons could be restored by forced expression of p27^kip1^, while simultaneously suppressing *Rnd2* expression via RNAi. Furthermore, the defective migration of *Rp58*-deficient cells within the embryonic cortex could be corrected by suppressing RhoA signalling by forced expression of a dominant-negative (N19) variant. Based on our results, we thus conclude that the co-presence of Rp58 and p27^kip1^ in embryonic cortical cells drives radial migration through parallel mechanisms which converge to suppress RhoA signalling. Notably, our results are consistent with the notion that RhoA signalling requires distinct upstream triggers [25, 36–38], since p27^kip1^ cannot substitute for the defective migration and cell morphological properties of *Rp58*-deficient neurons within the CP. Taken altogether, our results therefore raise the hypothesis for a dual-signalling cascade involving Rp58-p27^kip1^ and Rp58-Rnd2 pathways which, in turn, co-ordinately suppress RhoA signalling so as to modulate the neuronal cytoskeleton and drive efficient radial migration into the CP. As such, our study provides a compelling account of the molecular interplay between transcription factors and cyclin dependent kinase inhibition to promote cell cycle exit and radial migration during the development of the cerebral cortex. Future work will address the subcellular regulation of RhoA signalling which underlies the functional cooperativity of Rp58 and p27^kip1^ in these critical cellular processes for the assembly of functional neural circuits in the central nervous system.

## Additional files

Additional file 1: Figure S1. Steady state levels of p27^kip1^ are not significantly altered in Rp58^(+/−)^ and Rp58^(−/−)^ mutant mice compared to Rp58^(+/+)^ wildtype littermate controls. (A) Western blotting of mouse brain lysates from E14.5 mouse embryonic cortices of wildtype, heterozygote and homozygote mutant *Rp58*-knockout mice. Quantification was conducted on biological triplicates. (B) There was no significant difference in the steady state levels of immunoblotted p27^kip1^ signal (F_2,7_ = 0.26, *p* = 0.77, One-way ANOVA). (C) Analysis of p27^kip1^ immunofluorescence signal (red) in E14.5-electroporated, GFP-expressing cortical cells collected 36 h after surgical manipulation. Boxed inserts are representative of individual cells analysed from the IZ (C′) and VZ/SVZ (C″). (D) Raw images were analysed using ImageJ software to measure the average intensity of p27^kip1^ signal in GFP-labelled, IZ cells, with values in Arbitrary Units defined as the signal intensity of an IZ cell divided by the average intensity of 20 CP cells which are negative for GFP immunostaining, and multiplied by 100. There was no significant interaction between treatment groups (F_2,222_ = 2.9, *p* = 0.0557, One-way ANOVA, >75 cells counted from 3 independent brains per condition). (E) The average intensity of p27^kip1^ signal in GFP-labelled, VZ/SVZ cells, with values in Arbitrary Units defined as the signal intensity of an IZ cell divided by the average intensity of unelectroporated CP cells, and multiplied by 100. The intensity of p27^kip1^-immunofluorescence signal within VZ/SVZ cells was significantly reduced upon knockdown, while overexpression did not have a significant effect (F_2,222_ = 95, *p* < 0.0001, One-way ANOVA, >75 cells counted from 3 independent brains per condition). Values in (B), (D) and (E) represent mean ± SEM. Scale bars represent 100 μm (C) and 20 μm (C″) respectively. (PDF 208 kb)

Additional file 2: Figure S2. p27^kip1^ is a potent mediator of cell cycle exit and radial migration within the embryonic cortex. *In utero* electroporation of a control (GFP only), p27^kip1^ or p27^kip1^(ck-) construct into E14.5 embryonic cortices collected 36 h after surgery for analysis. (A-B) Sections were immunostained for Ki67 and counted to find that forced expression of p27^kip1^ but not p27^kip1^(ck-) led to a significant suppression in Ki67 co-staining (F_2,6_ = 53, *p* = 0.0002). (C-D) Sections were immunostained for pHH3, a marker of cell mitosis to find that forced expression of p27^kip1^ but not p27^kip1^(ck-) led to a significant suppression in pHH3 co-staining (F_2,6_ = 56, *p* = 0.0001). (E) Overexpression of p27^kip1^ or p27^kip1^ck- alone did not influence Pax6-immunoreactive radial glial progenitors (F_2,7_ = 0.35, *p* = 0.7171, One-way ANOVA, >600 cells counted from 3 to 4 independent brains per condition). (F) Treatment with *Rp58* siRNAs led to a significant reduction in the proportion of Tbr2-expressing intermediate progenitors which could not be augmented by co-delivery of p27^kip1^ or p27^kip1^ck- expression constructs (F_2,7_ = 11, *p* = 0.0072, One-way ANOVA, >600 cells counted from 3 to 4 independent brains per condition). (G) The distribution of GFP-labelled cells is significantly affected by treatment with p27^kip1^ or p27^kip1^(ck-). Forced expression of p27^kip1^ or p27^kip1^(ck-) led to a significant increase in the proportion of treated cells arriving within the upper IZ (F_8,30_ = 18, *p* < 0.0001; Two-way ANOVA followed by Bonferroni’s posthoc *t*-test; *p < 0.05, **p < 0.01, ***p < 0.001). Values in graphs represent mean ± SEM. Scale bars represent 50 μm. (PDF 428 kb)

Additional file 3: Figure S3. The effects of *Rp58* siRNA treatment together with expression constructs for p27^kip1^ and p27^kip1^ck- on progenitors. (A) There was a significant interaction between non-surface (SVZ) divisions in treated cells, identified as mitoses marked by pHH3 expression away from the ventricular surface (F_3,9_ = 7, *p* < 0.0102, One-way ANOVA, 3 independent brains per condition). (B-E) Representative photomicrographs of immunostained sections from each treatment, as indicated. (F) The proportion of Pax6-expressing radial glial progenitors was not significantly affected between treatment groups (F_3,9_ = 0.89, *p* = 0.4818, One-way ANOVA, >600 cells counted from 3 to 4 independent brains per condition). (G-J) Representative photomicrographs of GFP and Tbr2 immunostained sections from each treatment, as indicated. (K) The proportion of Tbr2-expressing intermediate progenitors was significantly affected between treatment groups (F_3,11_ = 6.5, *p* = 0.00858, One-way ANOVA, >600 cells counted from 3 to 4 independent brains per condition). Values in graphs represent mean ± SEM. Scale bars represent 50 μm. (PDF 193 kb)

Additional file 4: Figure S4. Immunostaining for Nestin reveals radial glial fibres within sections of electroporated (E14.5 + 72 h) mouse brains. Nestin immunostaining indicates that the radial glial scaffolds within the cortices of each treatment group are not qualitatively different. Scale bar represents 50 μm. (PDF 6040 kb)

Additional file 5: Figure S6. A summary diagram highlighting the combinatorial functions for Rp58 and p27^kip1^ on cell cycle exit and radial migration during the development of the mouse embryonic cerebral cortex. In this scheme, the regulation of neuroprogenitor cell cycle exit and neurogenesis is mediated by Rp58. Newborn postmitotic cells within the E14.5 embryonic cortex undergo cell cycle exit and express p27^kip1^ and Neurog2. The timing of p27^kip1^ expression and Neurog2 expression is influenced by Rp58. Neurog2 stimulates the expression of *Rp58* which induces a feedback loop to temper *Neurog2* expression levels, as well as to abrogate *Rnd2* expression in migrating cortical neurons. Meanwhile, p27^kip1^ stabilises Neurog2 protein levels to specify glutamatergic neuron identity as well as promote radial migration. In the context of *Rp58* deficiency, cortical cells lose their capacity to transit from the IZ to the CP owing to their failure to undergo MP to BP transition (B). Neither forced expression of p27^kip1^ nor *Rnd2* RNAi alone was capable of restoring the capacity of *Rp58*-deficient cells to migrate into the CP (C-D). However, the defective migration of *Rp58*-deficient cells was significantly augmented by the combination of p27^kip1^ overexpression and *Rnd2* RNAi (F). This restorative capacity is reminiscent of *Rp58*-deficient cells co-treated with RhoA(N19), a dominant-negative form which suppresses RhoA signalling (see Fig. 4). (PDF 123 kb)

Additional file 6: Figure S5. Forced expression of p27^kip1^ enhances radial migration and alters the morphological characteristics of embryonic cortical neurons. (A-B) Forced expression of p27^kip1^ leads to enhanced radial migration, observed as a significant increase in the proportion of cells arriving in the CP (F_2,12_ = 7.245, *p* = 0.0085; Two-way ANOVA followed by Bonferroni’s posthoc *t*-test; *p < 0.05) (C). (D) The intracortical positioning of p27^kip1^-overexpressing cells is also affected, observed as a significant increase in the proportion of cells within the upper CP (uCP) (F_2,12_ = 2.645, *p* = 0.118; Two-way ANOVA followed by Bonferroni’s posthoc *t*-test; *p < 0.05). (E-F) Representative images of IZ neurons within each treatment condition. (G) The distribution of cell shapes within the IZ are not significantly affected by p27^kip1^ overexpression (F_2,24_ = 1.8, *p* = 0.194; Two-way ANOVA. (H-J) Representative images of CP neurons within each treatment condition. Pie charts representing the lengths of the leading processes of uni/bipolar shaped neurons within the CP are represented for each treatment condition. (J) There is a significant interaction between the distribution of CP cell shapes and p27^kip1^ overexpression (F_2,27_ = 5.0, *p* = 0.0147; Two-way ANOVA), but the differences for a given cell shape was not significantly different upon posthoc *t*-testing. (K) The leading processes of p27^kip1^-overexpressing neurons were significantly shorter when compared with control (*p* < 0.0001 student’s *t*-test, two-tailed; *n* > 285 neurons per condition). Scale bars represent 100 μm (B) and 20 μm (F, I) respectively. (PDF 331 kb)

## Abbreviations

CDKI: Cyclin dependent kinase inhibitor
CP: Cortical plate
GFP: Green Fluorescent Protein
IZ: Intermediate zone
lCP: Lower cortical plate
mCP: Medial cortical plate
Rp58: Repressor Protein of 58 kDa
siRNA: Small inhibitory RNA
SVZ: Subventricular zone
uCP: Upper cortical plate
VZ: Ventricular zone
