# Correction to: Rp58 and p27^kip1^ coordinate cell cycle exit and neuronal migration within the embryonic mouse cerebral cortex

**DOI:** 10.1186/s13064-017-0098-x

**Published:** 2018-01-11

**Authors:** Olivier Clément, Isabel Anne Hemming, Ivan Enghian Gladwyn-Ng, Zhengdong Qu, Shan Shan Li, Michael Piper, Julian Ik-Tsen Heng

**Affiliations:** 1 The Harry Perkins Institute of Medical Research, Perth, WA 6009 Australia; 2 The Centre for Medical Research, University of Western Australia, Perth, WA 6009 Australia; 3 EMBL Australia, The Australian Regenerative Medicine Institute, Monash University, Clayton, VIC 3800 Australia; 4 The School of Biomedical Sciences, University of Queensland, Brisbane, 4072 Australia; 5 Queensland Brain Institute, University of Queensland, Brisbane, 4072 Australia; 6 Curtin Health Innovation Research Institute, Curtin University, Bentley, 6845 Australia

## Correction

After publication of the original article [1] it was realised that there were errors in figures 2a,b,f,g, which arose as a result of preparing figures from data collected and analysed at the same time as the work reported in [2] (Supplementary Figure 1 of [2]).

An updated Fig. 2 is included with this Correction.

**Fig. 2:**
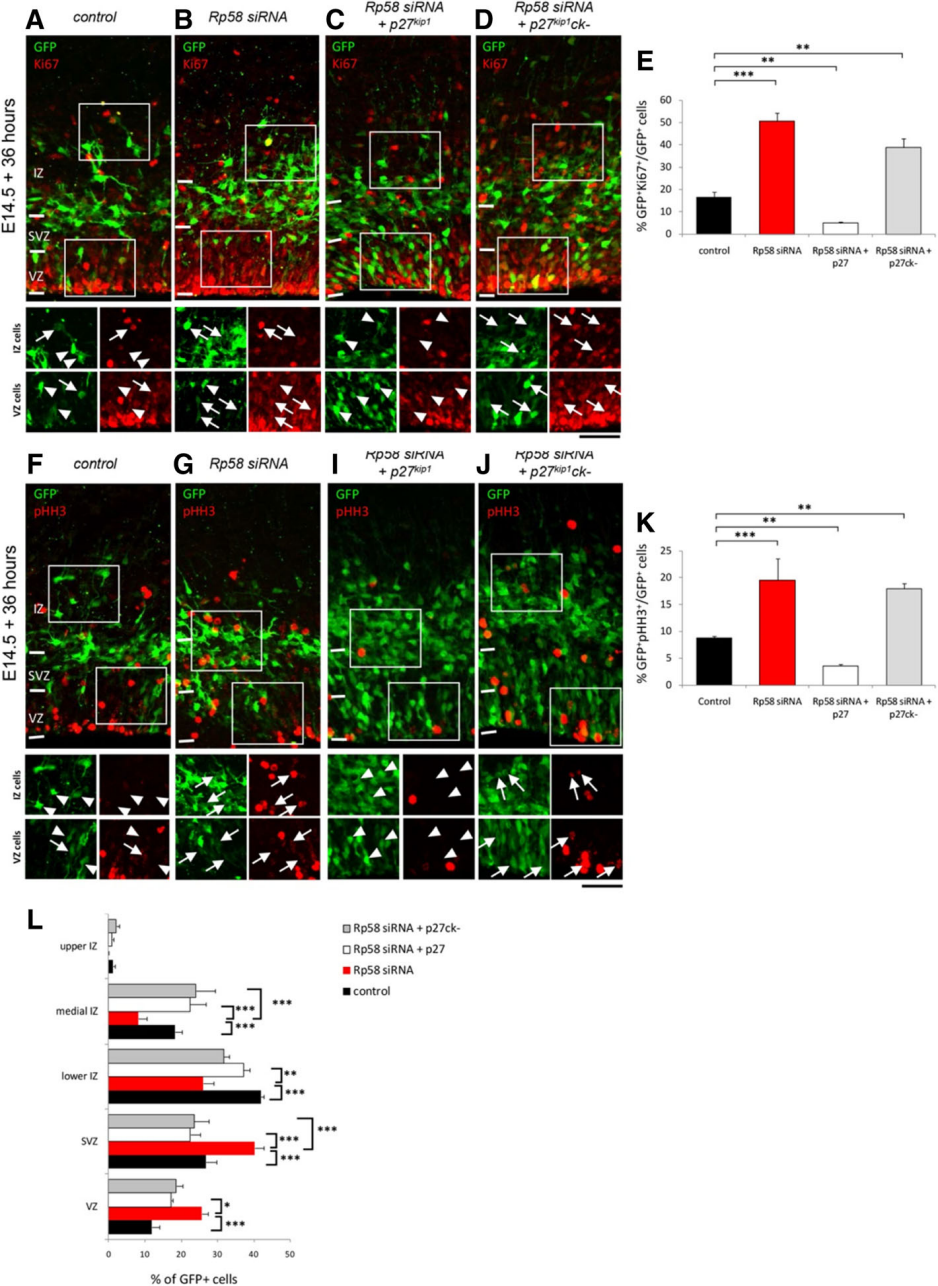
p27kip1 restores the defective cell proliferation and radial migration of Rp58 siRNA-treated cortical progenitors. Knockdown of *Rp58* leads to a significant reduction in the expression of the cell proliferation marker Ki67. **a–d** The defective expression of Ki67 in *Rp58* siRNA-treated cells could be restored with p27kip1, but not p27kip1(ck-) which is incapable of signalling cell cycle exit owing to a mutation which impairs its cyclin kinase function (**e**) (F3,8 = 73, *p* < 0.001, One-way ANOVA, >700 cells counted from 3 independent brains per condition). Similar effects on the co-detection of pHH3, a marker of cell mitosis, were observed (**f–k**, F2,8 = 20, *p* = 0.004, One-way ANOVA, >700 cells counted from 3 independent brains per condition). **l** In addition, suppression of *Rp58* by siRNA treatment impaired the migration of GFP-labelled cells, while treatment with either p27kip1 or p27kip1(ck-) promoted the radial migration of *Rp58*-siRNA treated cells from the VZ/SVZ to the IZ (F2,8 = 12, *p* < 0.0001, One-way ANOVA, >550 cells counted from 3 independent brains per condition). Scale bar represents 50 μm
