# Supplementary figures and images for: Rp58 and p27kip1 coordinate cell cycle exit and neuronal migration within the embryonic mouse cerebral cortex
Source: Neural Dev. 2017 May 15;12:8. doi: 10.1186/s13064-017-0084-3 (PMC5433244; doi:10.1186/s13064-017-0084-3)

Supplementary Figure S1

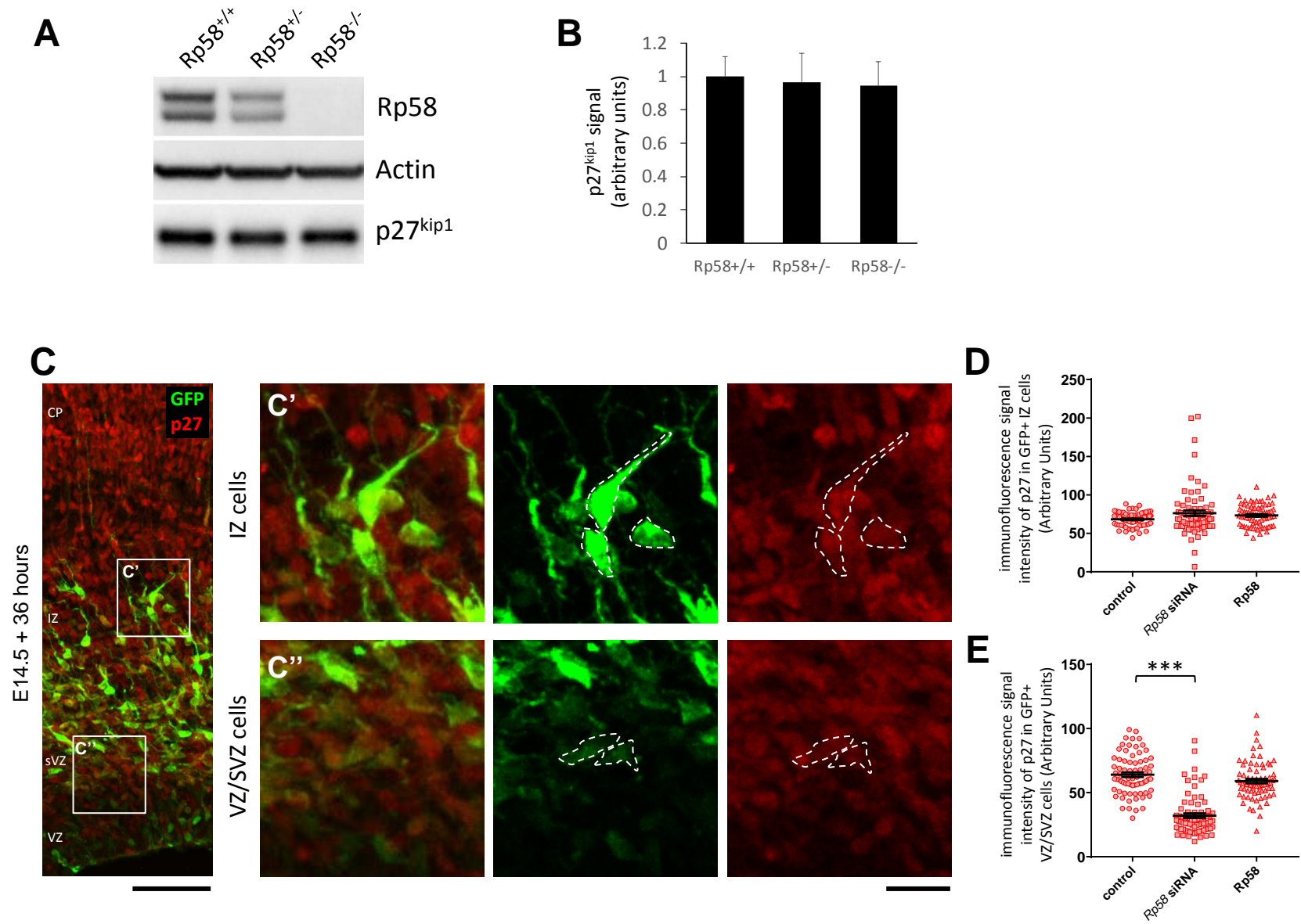

Supplement: Supplementary file 1 — Steady state levels of p27kip1 are not significantly altered in Rp58(+/−) and Rp58(−/−) mutant mice compared to Rp58(+/+) wildtype littermate controls. (A) Western blotting of mouse brain lysates from E14.5 mouse embryonic cortices of wildtype, heterozygote and homozygote mutant Rp58-knockout mice. Quantification was conducted on biological triplicates. (B) There was no significant difference in the steady state levels of immunoblotted p27kip1 signal (F2,7 = 0.26, p = 0.77, One-way ANOVA). (C) Analysis of p27kip1 immunofluorescence signal (red) in E14.5-electroporated, GFP-expressing cortical cells collected 36 h after surgical manipulation. Boxed inserts are representative of individual cells analysed from the IZ (C′) and VZ/SVZ (C″). (D) Raw images were analysed using ImageJ software to measure the average intensity of p27kip1 signal in GFP-labelled, IZ cells, with values in Arbitrary Units defined as the signal intensity of an IZ cell divided by the average intensity of 20 CP cells which are negative for GFP immunostaining, and multiplied by 100. There was no significant interaction between treatment groups (F2,222 = 2.9, p = 0.0557, One-way ANOVA, >75 cells counted from 3 independent brains per condition). (E) The average intensity of p27kip1 signal in GFP-labelled, VZ/SVZ cells, with values in Arbitrary Units defined as the signal intensity of an IZ cell divided by the average intensity of unelectroporated CP cells, and multiplied by 100. The intensity of p27kip1-immunofluorescence signal within VZ/SVZ cells was significantly reduced upon knockdown, while overexpression did not have a significant effect (F2,222 = 95, p < 0.0001, One-way ANOVA, >75 cells counted from 3 independent brains per condition). Values in (B), (D) and (E) represent mean ± SEM. Scale bars represent 100 μm (C) and 20 μm (C″) respectively. (PDF 208 kb) [file 13064_2017_84_MOESM1_ESM.pdf]

Supplementary Figure S2

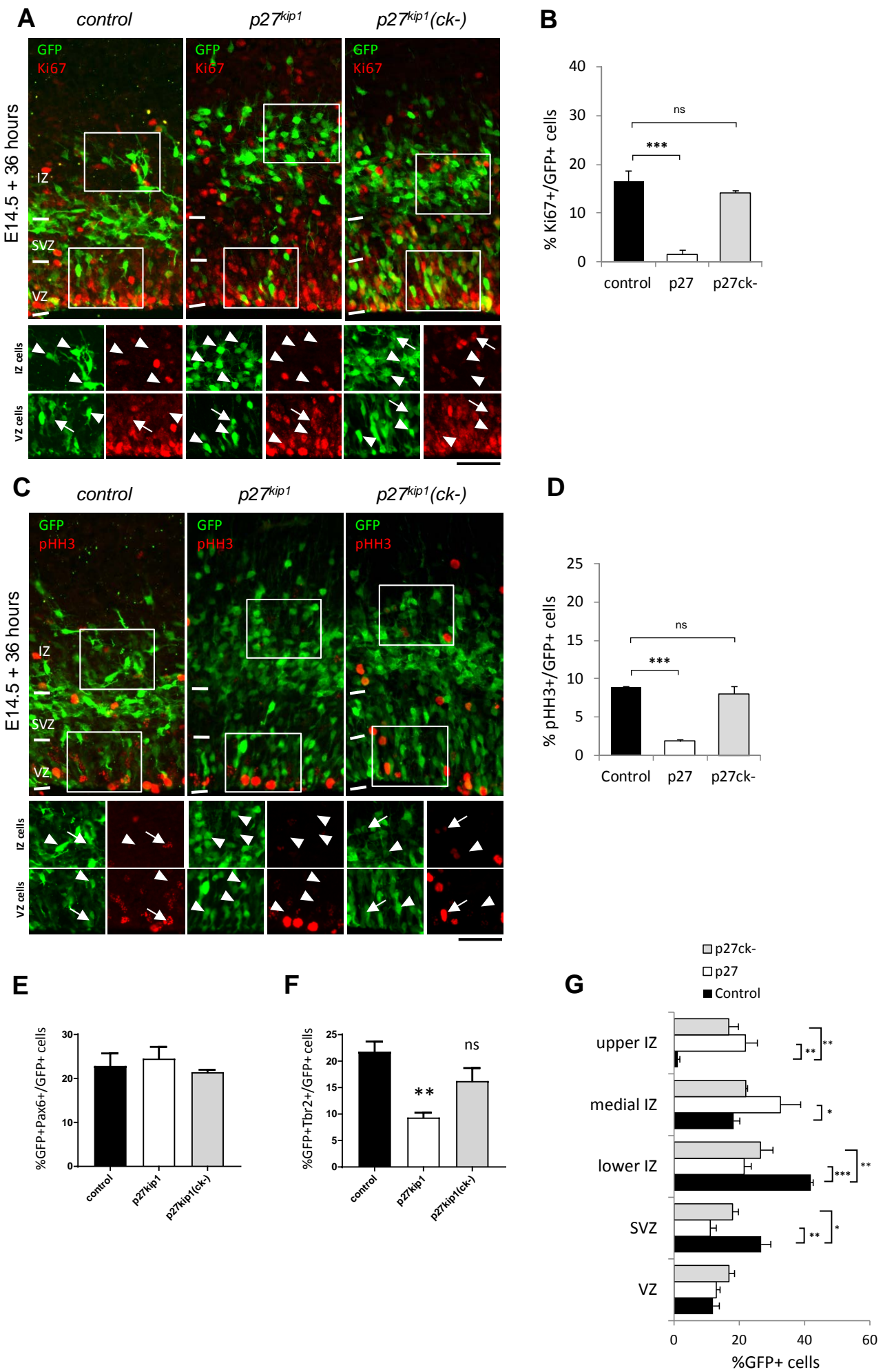

Supplement: Supplementary file 2 — p27kip1 is a potent mediator of cell cycle exit and radial migration within the embryonic cortex. In utero electroporation of a control (GFP only), p27kip1 or p27kip1(ck-) construct into E14.5 embryonic cortices collected 36 h after surgery for analysis. (A-B) Sections were immunostained for Ki67 and counted to find that forced expression of p27kip1 but not p27kip1(ck-) led to a significant suppression in Ki67 co-staining (F2,6 = 53, p = 0.0002). (C-D) Sections were immunostained for pHH3, a marker of cell mitosis to find that forced expression of p27kip1 but not p27kip1(ck-) led to a significant suppression in pHH3 co-staining (F2,6 = 56, p = 0.0001). (E) Overexpression of p27kip1 or p27kip1ck- alone did not influence Pax6-immunoreactive radial glial progenitors (F2,7 = 0.35, p = 0.7171, One-way ANOVA, >600 cells counted from 3 to 4 independent brains per condition). (F) Treatment with Rp58 siRNAs led to a significant reduction in the proportion of Tbr2-expressing intermediate progenitors which could not be augmented by co-delivery of p27kip1 or p27kip1ck- expression constructs (F2,7 = 11, p = 0.0072, One-way ANOVA, >600 cells counted from 3 to 4 independent brains per condition). (G) The distribution of GFP-labelled cells is significantly affected by treatment with p27kip1 or p27kip1(ck-). Forced expression of p27kip1 or p27kip1(ck-) led to a significant increase in the proportion of treated cells arriving within the upper IZ (F8,30 = 18, p < 0.0001; Two-way ANOVA followed by Bonferroni’s posthoc t-test; *p < 0.05, **p < 0.01, ***p < 0.001). Values in graphs represent mean ± SEM. Scale bars represent 50 μm. (PDF 428 kb) [file 13064_2017_84_MOESM2_ESM.pdf]

Supplementary Figure S3

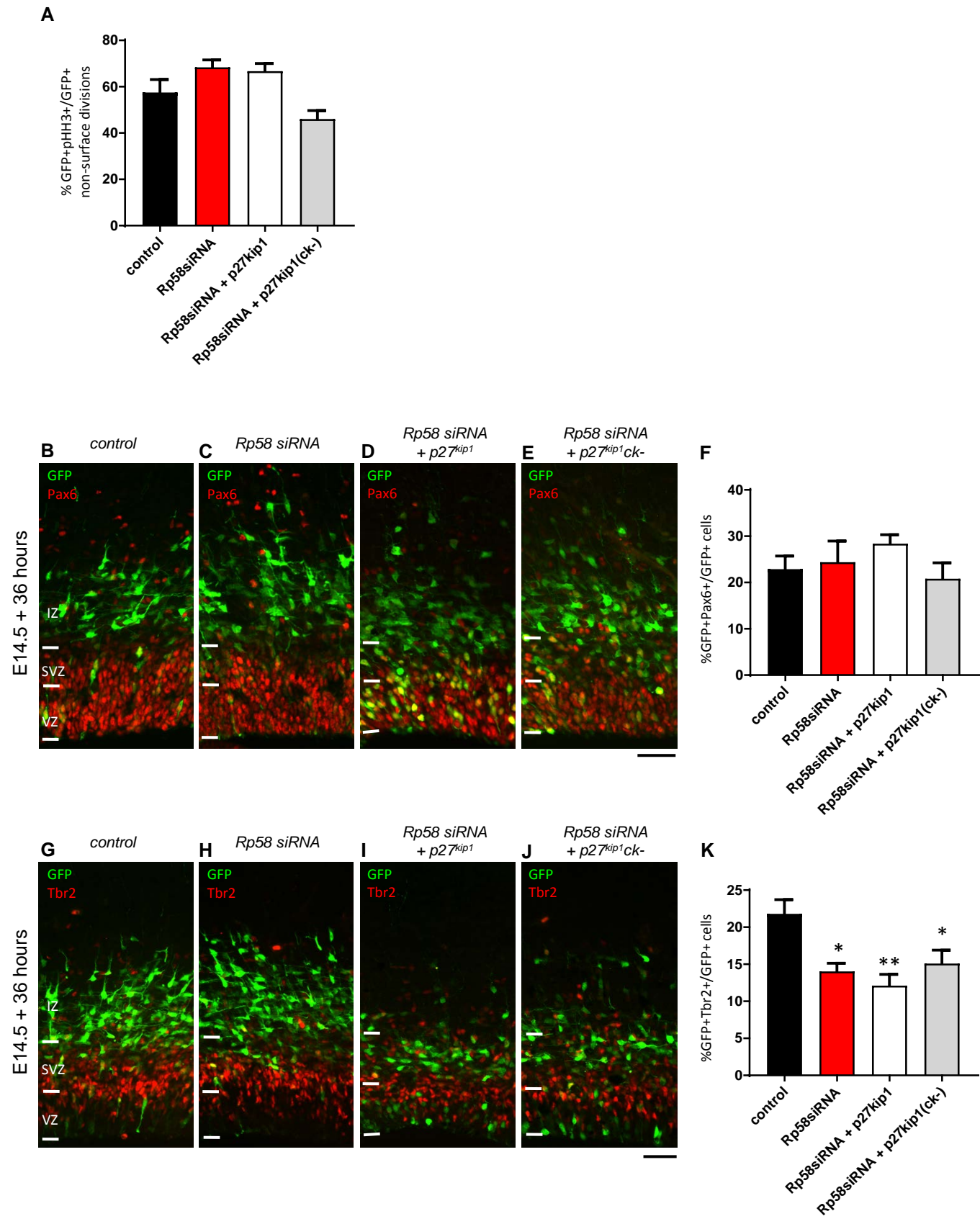

Supplement: Supplementary file 3 — The effects of Rp58 siRNA treatment together with expression constructs for p27kip1 and p27kip1ck- on progenitors. (A) There was a significant interaction between non-surface (SVZ) divisions in treated cells, identified as mitoses marked by pHH3 expression away from the ventricular surface (F3,9 = 7, p < 0.0102, One-way ANOVA, 3 independent brains per condition). (B-E) Representative photomicrographs of immunostained sections from each treatment, as indicated. (F) The proportion of Pax6-expressing radial glial progenitors was not significantly affected between treatment groups (F3,9 = 0.89, p = 0.4818, One-way ANOVA, >600 cells counted from 3 to 4 independent brains per condition). (G-J) Representative photomicrographs of GFP and Tbr2 immunostained sections from each treatment, as indicated. (K) The proportion of Tbr2-expressing intermediate progenitors was significantly affected between treatment groups (F3,11 = 6.5, p = 0.00858, One-way ANOVA, >600 cells counted from 3 to 4 independent brains per condition). Values in graphs represent mean ± SEM. Scale bars represent 50 μm. (PDF 193 kb) [file 13064_2017_84_MOESM3_ESM.pdf]

Supplementary Figure S4

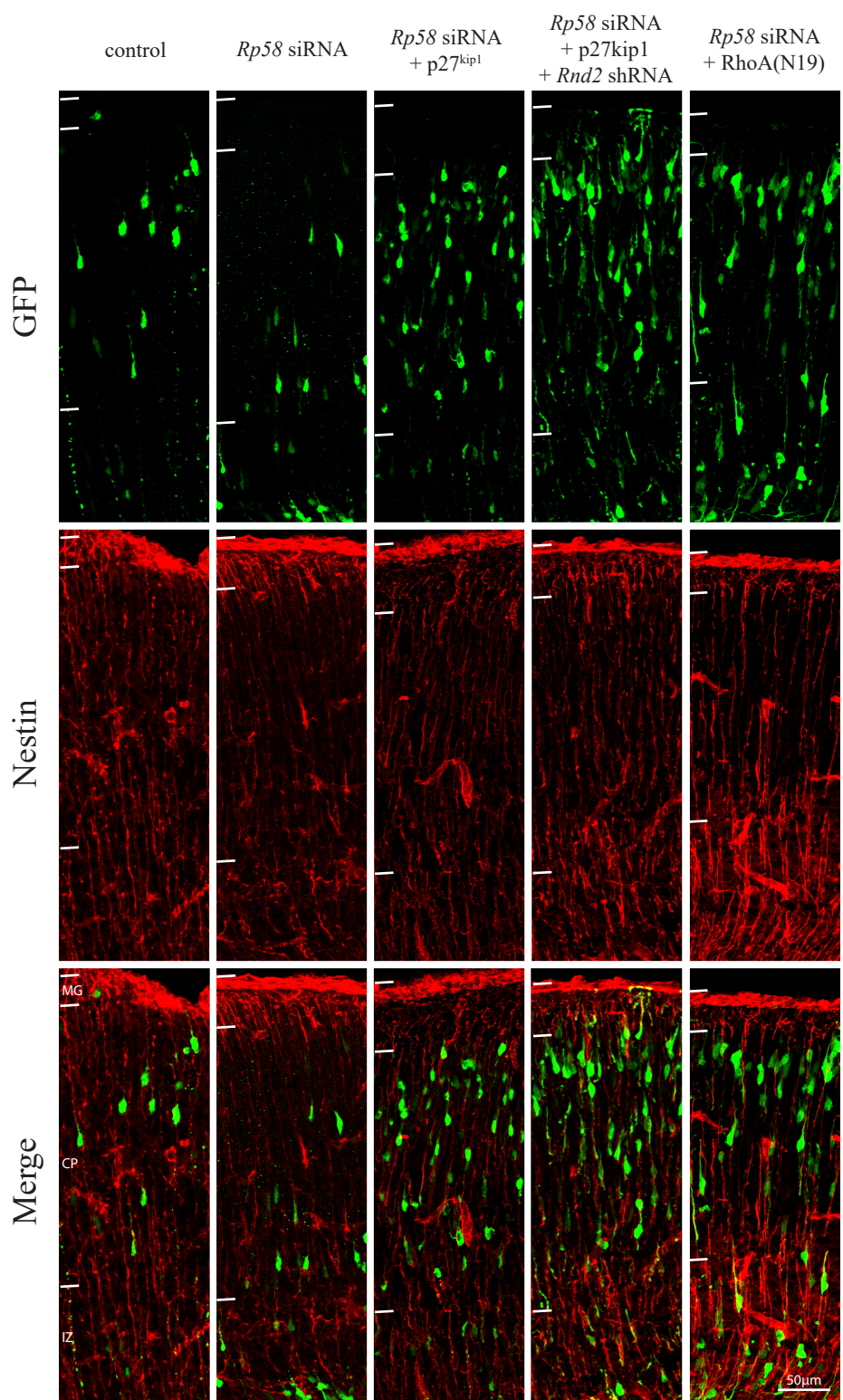

Supplement: Supplementary file 4 — Immunostaining for Nestin reveals radial glial fibres within sections of electroporated (E14.5 + 72 h) mouse brains. Nestin immunostaining indicates that the radial glial scaffolds within the cortices of each treatment group are not qualitatively different. Scale bar represents 50 μm. (PDF 6040 kb) [file 13064_2017_84_MOESM4_ESM.pdf]

Supplementary Figure S6

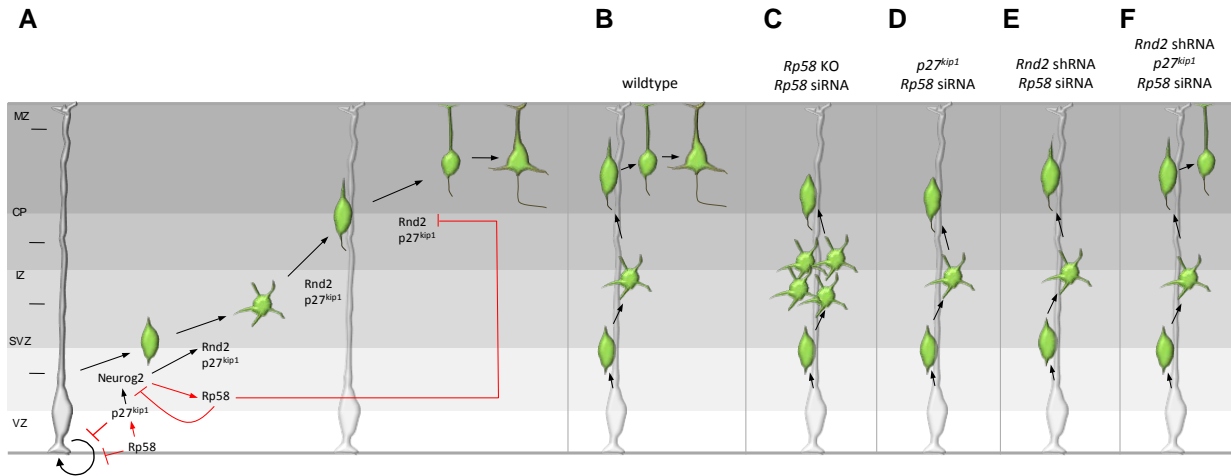

Supplement: Supplementary file 5 — A summary diagram highlighting the combinatorial functions for Rp58 and p27kip1 on cell cycle exit and radial migration during the development of the mouse embryonic cerebral cortex. In this scheme, the regulation of neuroprogenitor cell cycle exit and neurogenesis is mediated by Rp58. Newborn postmitotic cells within the E14.5 embryonic cortex undergo cell cycle exit and express p27kip1 and Neurog2. The timing of p27kip1 expression and Neurog2 expression is influenced by Rp58. Neurog2 stimulates the expression of Rp58 which induces a feedback loop to temper Neurog2 expression levels, as well as to abrogate Rnd2 expression in migrating cortical neurons. Meanwhile, p27kip1 stabilises Neurog2 protein levels to specify glutamatergic neuron identity as well as promote radial migration. In the context of Rp58 deficiency, cortical cells lose their capacity to transit from the IZ to the CP owing to their failure to undergo MP to BP transition (B). Neither forced expression of p27kip1 nor Rnd2 RNAi alone was capable of restoring the capacity of Rp58-deficient cells to migrate into the CP (C-D). However, the defective migration of Rp58-deficient cells was significantly augmented by the combination of p27kip1 overexpression and Rnd2 RNAi (F). This restorative capacity is reminiscent of Rp58-deficient cells co-treated with RhoA(N19), a dominant-negative form which suppresses RhoA signalling (see Fig. 4). (PDF 123 kb) [file 13064_2017_84_MOESM5_ESM.pdf]

Supplementary Figure S5

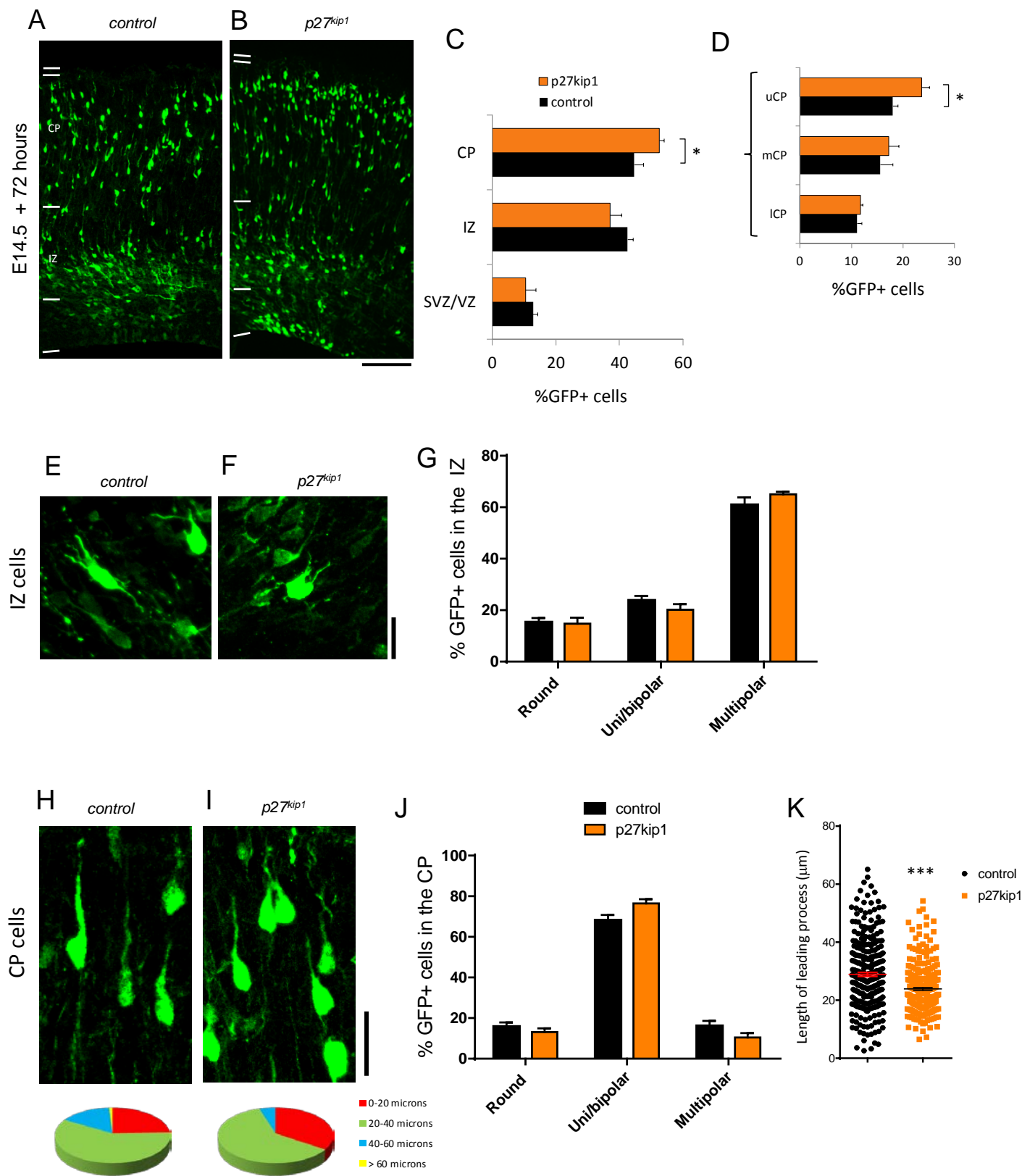

Supplement: Supplementary file 6 — Forced expression of p27kip1 enhances radial migration and alters the morphological characteristics of embryonic cortical neurons. (A-B) Forced expression of p27kip1 leads to enhanced radial migration, observed as a significant increase in the proportion of cells arriving in the CP (F2,12 = 7.245, p = 0.0085; Two-way ANOVA followed by Bonferroni’s posthoc t-test; *p < 0.05) (C). (D) The intracortical positioning of p27kip1-overexpressing cells is also affected, observed as a significant increase in the proportion of cells within the upper CP (uCP) (F2,12 = 2.645, p = 0.118; Two-way ANOVA followed by Bonferroni’s posthoc t-test; *p < 0.05). (E-F) Representative images of IZ neurons within each treatment condition. (G) The distribution of cell shapes within the IZ are not significantly affected by p27kip1 overexpression (F2,24 = 1.8, p = 0.194; Two-way ANOVA. (H-J) Representative images of CP neurons within each treatment condition. Pie charts representing the lengths of the leading processes of uni/bipolar shaped neurons within the CP are represented for each treatment condition. (J) There is a significant interaction between the distribution of CP cell shapes and p27kip1 overexpression (F2,27 = 5.0, p = 0.0147; Two-way ANOVA), but the differences for a given cell shape was not significantly different upon posthoc t-testing. (K) The leading processes of p27kip1-overexpressing neurons were significantly shorter when compared with control (p < 0.0001 student’s t-test, two-tailed; n > 285 neurons per condition). Scale bars represent 100 μm (B) and 20 μm (F, I) respectively. (PDF 331 kb) [file 13064_2017_84_MOESM6_ESM.pdf]
